# Changes in Serum Immunoglobulin Levels Play as Predictors of Treatment Response and Prognosis in Pediatric Idiopathic Nephrotic Syndrome During the Remission Phase

**DOI:** 10.1002/iid3.70144

**Published:** 2025-01-27

**Authors:** Amin sadat Sharif, Naghmeh Nickravesh, Marzieh Heidarzadeh Arani, Mohammad Javad Azadchehr, Hossein Motedayyen

**Affiliations:** ^1^ Pediatric Nephrology Department, Hasheminejad Hospital Iran University of Medical Sciences Tehran Iran; ^2^ Pediatric Department, School of Medicine Kashan University of Medical Sciences Kashan Iran; ^3^ Infectious Diseases Research Center Kashan University of Medical Sciences Kashan Iran; ^4^ Autoimmune Diseases Research Center Kashan University of Medical Sciences Kashan Iran

**Keywords:** disease remission, idiopathic nephrotic syndrome, immune dysfunctions, immunoglobulins

## Abstract

**Background:**

Nephrotic syndrome is an immune‐mediated renal disorder characterized by T‐cell and B‐cell dysfunctions with changes in immunoglobulin (Ig) levels and the IgG:IgM ratio. Therefore, this study aimed to determine whether the serum level of Igs can be considered as an index to predict the response to treatment and the prognosis of idiopathic nephrotic syndrome (INS) in children in the remission phase.

**Methods:**

The study population consisted of 38 children with INS in the remission phase and 38 age‐ and sex‐matched healthy children. Blood samples were collected from participants and serum values of IgG, IgM, IgE, and IgA were measured using EISA KITS from Aptech Services. The IgG:IgM ratio was studied in the participants.

**Results:**

Patients significantly increased IgM and IgE levels compared with healthy subjects, unlike IgG and IgA values (*p* < 0.001–0.05). Patients with steroid‐resistant nephrotic syndrome (SRNS) had a significant increase in IgM levels compared with those with steroid‐sensitive nephrotic syndrome (SSNS) (*p* < 0.05). While, subjects with SRNS showed significant reductions in IgG and IgA values (*p* < 0.01). There were significant differences in the levels of IgG and IgM between steroid‐sensitive patients with and without a history of relapse (*p* < 0.01). Furthermore, patients with steroid‐independent and frequently relapsing NS showed a significant increase in IgE value compared with that of subjects with steroid‐dependent and relapse (*p* < 0.05). The ratio of IgG/IgM was significantly reduced in patients compared with healthy individuals (*p* < 0.05). Other results indicated that there was a significant difference between patients with steroid‐independent and steroid‐dependent who had a history of relapse (*p* < 0.01).

**Conclusion:**

Alterations in serum Ig values can be considered as predictors of treatment response and prognosis in pediatric idiopathic nephrotic syndrome during the remission phase.

## 1 Introduction

Nephrotic syndrome (NS) is characterized by severe proteinuria (40 mg/m^2^/h), hypoalbuminemia, and edema [1]. Different immune cells such as T and B cells may be stimulated idiopathically or by infections and allergens [2, 3, 4]. Cytokines secreted from stimulated immune cells damage the filtration barrier in the endothelium, basement membrane, podocyte, or a combination of these layers [5]. Most children with NS have the initial form of the disease, which includes the following: minimal change disease (most common), focal segmental glomerulosclerosis, membranoproliferative glomerulonephritis, membranous nephropathy, and diffuse mesangial proliferation. This disease can occur secondary to systemic diseases such as vasculitis, infections, and malignancies [6].

Idiopathic nephrotic syndrome (INS) is divided into steroid‐sensitive nephrotic syndrome (SSNS) and steroid‐resistant nephrotic syndrome (SRNS) based on their response to steroid therapy. Most children with SSNS have frequent relapses [7, 8]. Nearly 80%–90% of children respond to steroid treatment within 4 weeks, which includes clinical improvement, diuresis, and negative or brief urine in terms of protein in 3 consecutive days. If the patient has gone to the remission phase and now has proteinuria (≥ 3+) for 3 consecutive days, it means relapse. Children who have proteinuria (≥ 3+) after 8 weeks of steroid treatment are considered resistant to steroids and need a diagnostic kidney biopsy. A group of patients during treatment with steroids every other day or within 28 days after completing the treatment period. These patients are called steroid dependent. If the relapse of the disease occurs due to a stimulating factor such as infection, allergy, vaccine, and so forth, it means steroid independent [2, 9, 10, 11].

NS is an immune‐mediated kidney disease associated with T‐cell dysfunction and secondary B‐cell dysfunction that leads to changes in the level of immunoglobulins (Igs) [12]. The presence of T cells is not necessary for the development of B lymphocytes into mature B lymphocytes in the bone marrow, but the presence of T cells and their cytokines is necessary for the transformation of mature B lymphocytes into antibody‐producing plasma cells. In this case, the activated B cell undergoes switching and is able to convert IgM to other classes of heavy chains such as γ, α, or ε [9, 13, 14, 15].

T‐cell defects in NS lead to switching defects. Therefore, dysgammaglobulinemia (low IgG, but high IgM levels) is a common feature in most nephrotic patients [16]. In the studies that have been conducted in this field, data collection has been done in the active phase of the disease or a combination of active and remission. In the active phase of the disease, these patients are prone to hypogammaglobulinemia due to the urinary excretion of IgG and increased catabolism. In this study, for the first time, we decided to work on the nature of the physiopathology of the disease, which is why all patients are in the remission phase. Regarding that hypogammaglobulinemia makes patients susceptible to various infections, which itself triggers the relapse of nephrotic disease, therefore, our aim is the investigation of serum Ig levels in children with NS in the remission phase and their relationships with different types of the disease.

## 2 Materials and Methods

The present study is a cross‐sectional‐analytical study which was conducted from January 2022 to May 2023. A total of 38 children with INS were recruited among those referred to the pediatric clinics of Shahid Beheshti and Akhwan Hospitals, Kashan, Iran. The disease was diagnosed by a specialist according to the diagnostic criteria for NS [1, 17]. The initial steroid‐resistant nephrotic syndrome (ISRNS) and late steroid‐resistant nephrotic syndrome (LSRNS) were approved by a specialist as mentioned in previous studies [18, 19]. Thirty‐eight age‐ and sex‐matched healthy volunteers without any autoimmune abnormalities and health problems were considered as a control group. All patients were in the remission phase, while urine protein was negative or trace for 3 consecutive days. To classify the subjects into different groups, the patients were monitored for their response to treatment and the rate of relapse at least 1 year before study initiation. Exclusion criteria were: (1) secondary form of the disease; (2) severe malnutrition; (3) presence of protein‐losing enteropathies; (4) treatment with immunosuppressive drugs for other reasons; (5) immunodeficiency. Based on the type of INS in the remission phase, the patients were divided into two groups, including SSNS and SRNS groups. Furthermore, the SSNS group was divided into two subgroups; according to the presence and absence of relapse. The study and all protocols were approved by the Ethics Committee of Kashan University of Medical Sciences (reference number: IR.KAUMS.MEDNT.REC.1400.119). A questionnaire was used to collect demographic characteristics of the patients (age, sex, type of disease, duration of disease, and type of treatment).

### 2.1 Sample Collection and Antibody Assay

The blood samples (3 mL) were collected after obtaining informed consent from legally authorized representatives for volunteers. The liquid phase immunization methods were employed to assess the serum levels of IgG, IgM, IgA, and IgE levels using EISA KITS from Aptech Services.

### 2.2 Statistical Analysis

The GraphPad Prism 6 (GraphPad Software, USA) was employed to analyze the results. Data were represented as the mean ± standard deviation (SD) and mean ± standard error of the mean (SEM). The normal distributions of data were studied by the Kolmogorov–Smirnov test. The groups with the normal distributions were analyzed using one‐way analysis of variance and unpaired *t*‐tests, while those with non‐normal distributions were compared using Kruskal–Wallis and Mann–Whitney tests. *p* values ≤ 0.05 were considered statistically significant.

## 3 Results

### 3.1 Patient Descriptions

There was no significant difference between the two groups in terms of age and sex. Of the 38 patients with INS, 5 had steroid‐resistant and 33 showed steroid‐sensitive (Table 1). Of the five subjects with SRNS, one case showed ISRNS and four cases had LSRNS (Table 1). There was no significant difference in Ig levels between ISRNS and LSRNS individuals. Of the cases with steroid‐sensitive, 16 experienced relapses and 17 did not have. Of 17 cases without relapse, all patients were treated with prednisolone. Other demographics and clinical characteristics of participants are shown in Table 1.

**Table 1:** Demographic and clinical characteristics of participants.

	Healthy subjects (*n *= 38)		Subjects with INS (*n* = 38)		
Age, years (min–max)	5.44 ± 2.61 (1–18)		6.37 ± 4.95 (2–17)		
Gender (male/female)	18/20		23/15		
Ethnicity	Persian		Persian		
Age at onset, years (min–max)	—		5.33 ± 3.69 (1.3–15)		
Treatment	—		Prednisolone: 38 (100%) Mycophenolate mofetil: 9 (23.68%) Cyclosporine: 15 (39.47%) Rituximab: 5 (13.15%) Levamisole: 1 (2.63%)		
*Patients with INS undergoing the remission phase*					
SRNS	5 (13.2%)	ISRNS: 1 (20%) LSRNS: 4 (80%)			
SSNS	Relapse	Yes	16 (40.6%)	Steroid‐dependent	12 (31.6%)
				Steroid‐independent	4 (10.5%)
		No	17 (44.7%)	—	

Abbreviations: ISRNS, Initial steroid‐resistant nephrotic syndrome; LSRNS, late steroid‐resistant nephrotic syndrome; SRNS, steroid‐resistant nephrotic syndrome; SSNS, steroid‐sensitive nephrotic syndrome.

### 3.2 The Serum Levels of Antibodies in Patients With INS

**Figure 1:**
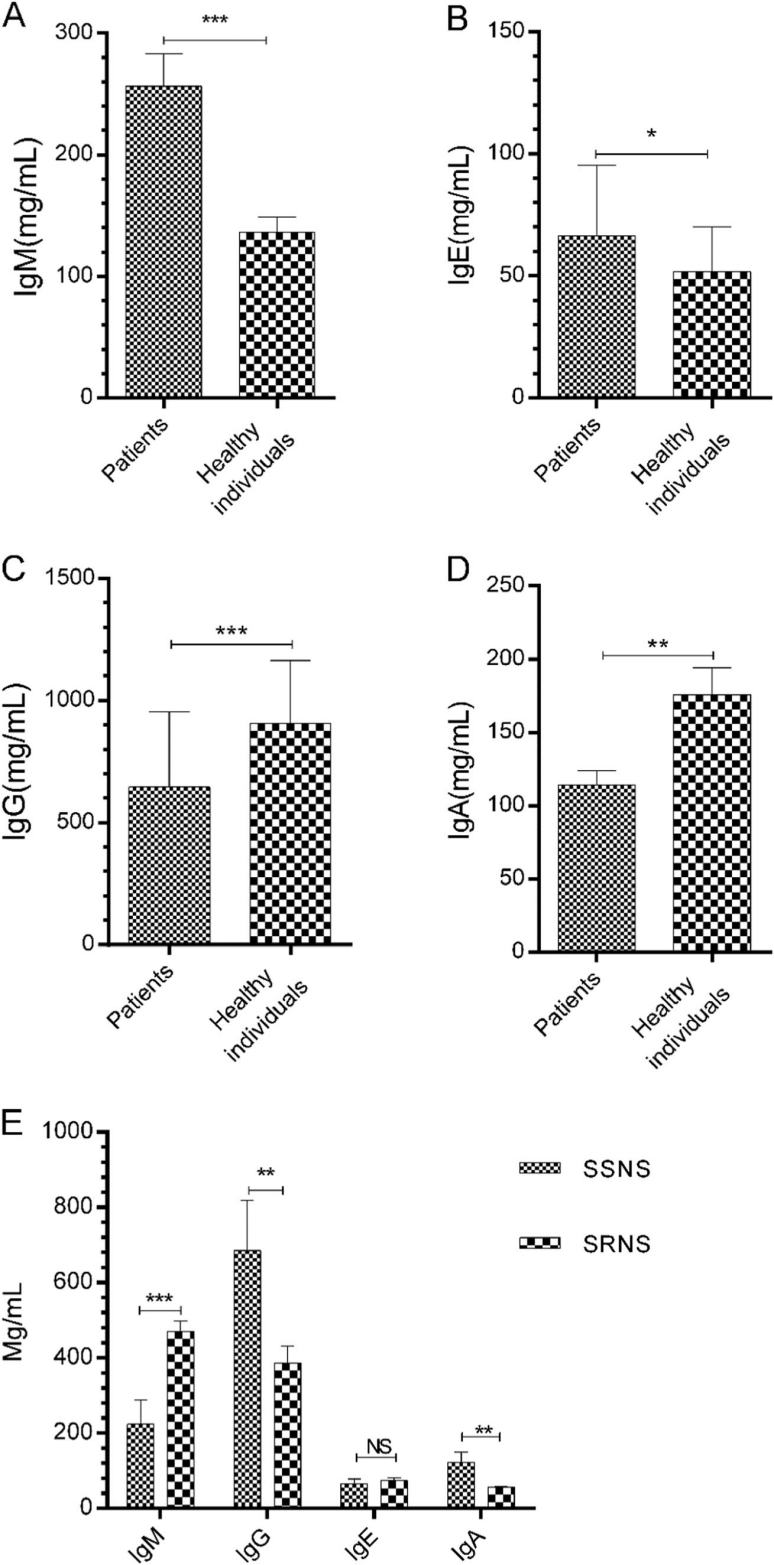
Serum antibody levels of patients with INS. (A–D) The serum levels of IgG, IgM, IgA, and IgE in patients (*n* = 38) and healthy subjects (*n* = 38) were measured using EISA KITS from Aptech Services. (E) The levels of antibodies were studied in patients with SRNS and SSNS. Data are shown as mean ± SD. *p < 0.05, **p < 0.01, ***p < 0.001.

The levels of IgG, IgM, IgA, and IgE in patients and healthy subjects were assessed. As shown in Figure 1 A,B, patients significantly increased IgM and IgE levels compared with healthy subjects (*p* < 0.001–0.05). Other results revealed that IgG and IgA were significantly lower in subjects with INS than the healthy group (*p* < 0.001–0.01, Figure 1C,D).

In the next step, the levels of antibodies were studied in patients with SSNS and SRNS. As shown in the Figure 1E, patients with steroid‐resistant had a significant increase in IgM level compared with that of those with steroid‐sensitive (*p* < 0.05). While, subjects with steroid resistance showed significant reductions in the values of IgG and IgA (*p* < 0.01, Figure 1E).

### 3.3 The Serum Levels of Antibodies in Patients With SSNS and Relapse

**Figure 2:**
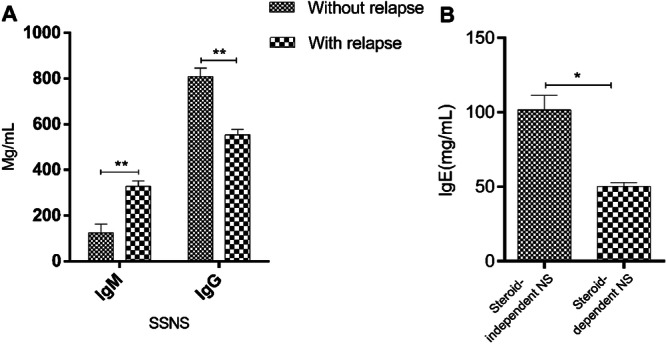
Serum antibody levels of SSNS patients with and without relapse. (A and B) The values of antibodies were studied in patients with SSNS and relapse. The results are shown as mean ± SD. *p < 0.05, **p < 0.01.

The values of antibodies were studied in patients with SSNS and frequently relapsing NS. Our data indicated that there were significant differences in the levels of IgG and IgM between patients with and without a history of relapse (*p* < 0.01, Figure 2A). Furthermore, patients with steroid‐independent and frequently relapsing NS showed a significant increase in IgE value compared with that of subjects with steroid‐dependent and relapse (*p* < 0.05, Figure 2B).

### 3.4 The Ratio of IgG/IgM in INS

**Figure 3:**
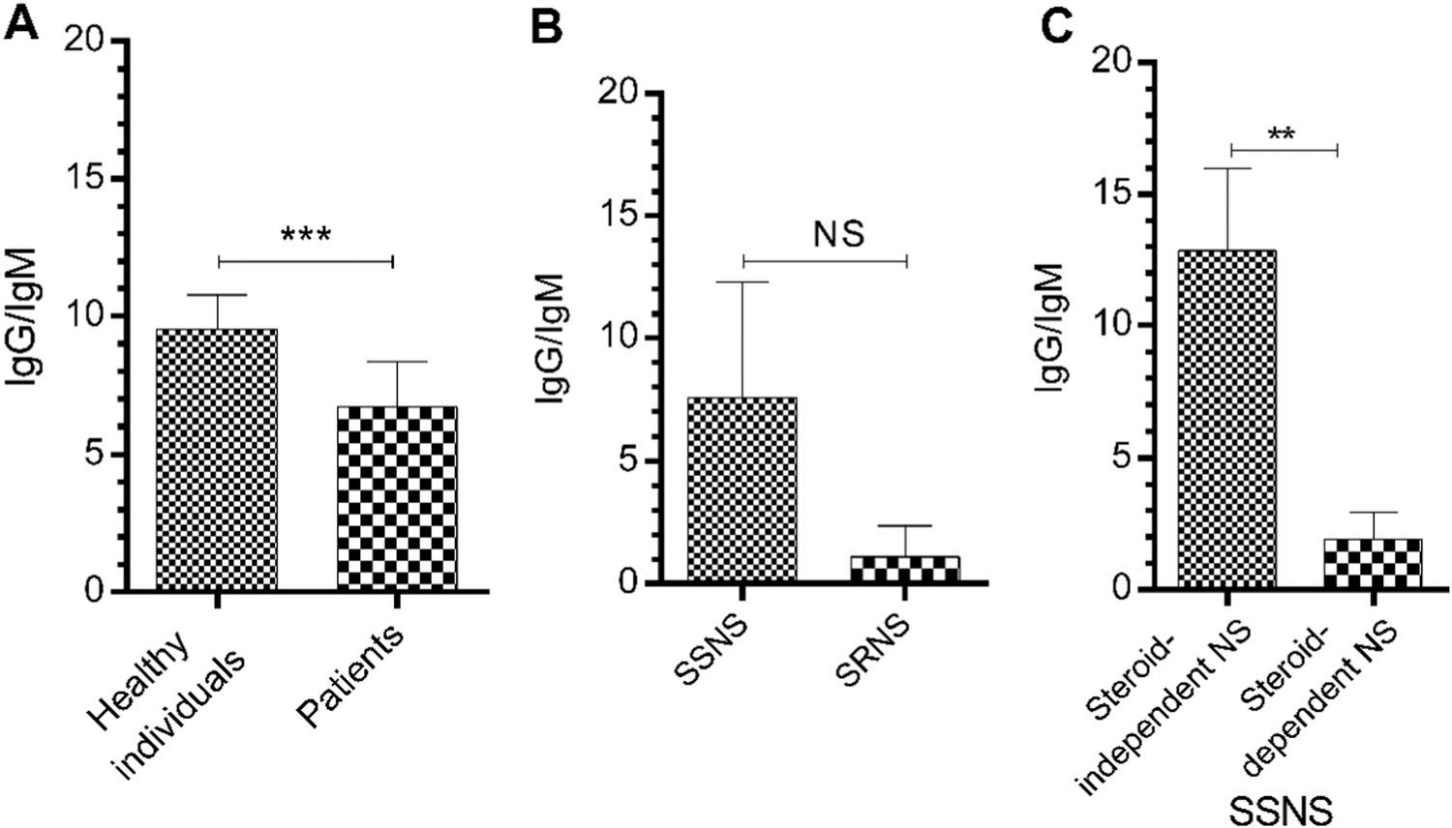
IgG/IgM ratio in healthy and patients with INS. (A) There was a significant difference between healthy subjects and patients, unlike subjects with SSNS and SRNS (B). (C) This difference was observed between SSNS patients with and without relapse. The results are shown as mean ± SD. **p < 0.01, ***p < 0.001.

Other results indicated that IgG/IgM ratio had a significant reduction in patients compared with healthy individuals (*p* < 0.05, Figure 3A). Although the ratio of IgG/IgM was lower in patients with SRNS than subjects with SSNS, this reduction was not statistically significant (Figure 3B). Other results indicated that there was a significant difference in IgG/IgM ratio between patients with steroid‐independent and steroid‐dependent who had a history of relapse (*p* < 0.01, Figure 3C).

## 4 Discussion

NS as an immune‐mediated kidney disease is associated with T‐ and B‐cell dysfunctions, leading to susceptibility to various infections and relapse [13]. Some reports have suggested that the measurements of IgG and IgM levels, the ratio of IgG/IgM, and their correlations with the clinical response to steroids, may be helpful in the evaluation of disease prognosis and treatment strategy [12]. Therefore, the current study investigated the serum levels of IgG, IgA, IgE, and IgM in children with INS who were in the remission phase of the disease.

To assess the immunological situation of patients with INS, the results revealed that patients in the remission phase of the disease had significant increases in IgM and IgE levels compared with healthy subjects. On the contrary, IgG and IgA were significantly lower in patients than in healthy subjects. The same trend was observed in patients with SRNS. Subjects with steroid‐resistant had a significant increase in IgM levels and notable reductions in IgG and IgA values, compared with those of individuals with steroid‐sensitive. These changes may be attributed to the defect in antibody class switching, due perhaps to impaired T‐cell function. There is an inconsistency in the literature regarding changes in Ig levels in patients with NS [20]. Some studies are consistent with this study showing the increased value of IgM accompanied by a reduction of IgG level [4, 21]. However, others have indicated the increased or normal production of IgG in patients with NS. The discrepancy observed in the literature may correlate to the measurement of antibody values in patients with different ages, numbers, and pathological types of NS [20].

In an effort to determine the values of antibodies in steroid‐sensitive patients who had the experience of relapse, their levels were studied. Our data indicated that patients with SSNS and relapse had a significant increase in IgM, unlike IgG, in comparison to patients without relapse. In line with this notion, previous studies have shown that the reduced level of serum IgG had a significant relationship with the relapse rate at the beginning of the disease [15]. Regarding the fact that NS is considered as an immune‐mediated disease with T‐ and B‐cell dysfunctions, relapse is largely related to susceptibility to various infections. Furthermore, some patients with SSNS and relapse were treated with rituximab, which plays a fundamental role in long‐term hypogammaglobulinemia and impaired immunity, especially in young children [22, 23, 24]. Therefore, it is likely that rituximab‐induced hypogammaglobulinemia participated in INS and susceptibility to various infections.

In the next step, the ratio of IgG/IgM in patients with INS under the remission phase was evaluated. Patients had a significant reduction in IgG/IgM ratio compared with healthy individuals. Although the ratio of IgG/IgM was lower in patients with SRNS than in subjects with SSNS, this reduction was not statistically significant. Other results indicated that there was a significant difference between SSNS patients with and without relapse [25].

Other findings for the first time indicated that IgE value was significantly higher in SSNS individuals with steroid‐independent and frequently relapsing NS than that of subjects with steroid‐dependent and relapse. The increased level of IgE in these patients may be associated with the presence of some stimulants such as allergens and infectious agents, leading to relapse.

Taken together, the results of the current study along with previous reports suggest that alterations of Ig values are related to the impaired function of T cells in antibody class switching and rituximab‐induced hypogammaglobulinemia. T‐cell dysfunction can lead to an increase in IgM level and reduction in other subtypes of antibodies, which contribute to susceptibility to infectious diseases and relapse. Defects in antibody class switching may occur during the remission phase of the disease with a greater severity in patients with steroid‐resistant. Therefore, the alterations of antibody levels and their ratio may act as a useful serological method to clarify treatment response and relapse during disease remission and lack of proteinuria. However, further studies with a larger sample size are needed to confirm our data.

## Author Contributions

**Amin sadat Sharif:** conceptualization, writing – original draft. **Naghmeh Nickravesh:** investigation. **Marzieh Heidarzadeh Arani:** investigation and disgn the experiments. **Mohammad Javad Azadchehr:** statistical analysis. **Hossein Motedayyen:** conceptualization and approving – original draft.

## Consent

Informed consent was taken before taking part in the study.

## Conflicts of Interest

The authors declare no conflicts of interest.

## Data Availability

The original contributions presented in the study are included in the article. Further inquiries can be directed to the corresponding authors.
